# Acute Gastrointestinal Bleed from a Gastrointestinal Stromal Tumour Impersonating an Arteriovenous Malformation of the Jejunum

**DOI:** 10.1155/2021/6674612

**Published:** 2021-01-18

**Authors:** Oshan Basnayake, Umesh Jayarajah, Nilesh Fernandopulle, Sivasuriya Sivaganesh

**Affiliations:** ^1^Professorial Surgical Unit, National Hospital of Sri Lanka, Colombo, Sri Lanka; ^2^Department of Surgery, Faculty of Medicine, University of Colombo, Sri Lanka

## Abstract

Gastrointestinal stromal tumours (GIST) are neoplasms which originate from the mesenchymal tissue of the gastrointestinal tract. We report on a GIST presenting with acute gastrointestinal bleeding that mimicked an arteriovenous malformation (AVM) on imaging and at surgery. A 61-year-old female presented with a short history of melaena and severe anaemia. After resuscitation, upper gastrointestinal endoscopy showed a profusely bleeding varix in the proximal jejunum which was treated with glue injection. Contrast-enhanced CT showed a heterogeneous lesion in the proximal jejunum with strong arterial phase enhancement, supplied by a branch of the superior mesenteric artery and drained by a prominent tributary of the superior mesenteric vein, suggestive of an AVM. The mass was resected, and histology revealed a GIST with no evidence of an AVM or angiodysplasia. A GIST may be considered, though imaging suggests a diagnosis of an AVM in patients presenting with acute gastrointestinal bleeds. In such circumstances, surgical resection and pathological assessment will be confirmatory.

## 1. Introduction

Gastrointestinal stromal tumours are a subset of neoplasms which originate from the mesenchymal tissue of the gastrointestinal tract. They differ from other mesenchymal neoplasms such as leiomyomas, leiomyosarcomas, and spindle cell tumours [1]. Reported incidence of GISTs ranges from 0.55 to 0.78 per 100,000 with a male predominance [2]. However, the presentation of a GIST mimicking an arteriovenous malformation (AVM) is rare and only few cases have been reported [3–6]. We describe a 61-year-old female with an unusual presentation of a GIST as an AVM.

## 2. Case Presentation

A 61-year-old female with hypertension and dyslipidaemia presented with the passage of altered blood of 4-day duration. This was the first episode, and her bowel habits had been normal to date. On admission, she was pale with a pulse rate of 118 per minute and a blood pressure of 86/66 mmHg. Her haemoglobin was 6.2 g/dl, and her coagulation profile, liver functions, and renal functions were normal.

**Figure 1:**
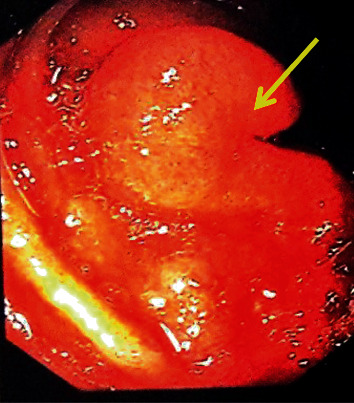
Yellow arrow shows the endoscopic appearance of bleeding varix at proximal jejunum.

**Figure 2:**
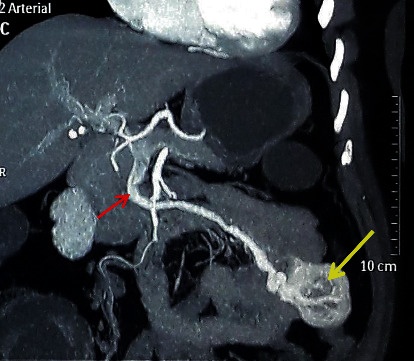
Coronal view of contrast-enhanced CT scan image arterial phase: yellow arrow shows the jejunal lesion; red arrow shows the contrast enhancement of superior mesenteric vein.

She was resuscitated initially with crystalloids and then packed red cell transfusions. The next day, she underwent upper gastrointestinal endoscopy (UGIE) which revealed a protruding mass with active bleeding in the distal duodenum. Upper gastrointestinal assessment was repeated in the same setting using a paediatric colonoscope, and a profusely bleeding varix was observed in the proximal jejunum (Figure 1). Haemostasis was achieved by glue injection. A colonoscopy was also performed in the same setting which was normal. Abdominal ultrasonography was unremarkable. On day 6 after admission, a contrast CT of the abdomen was performed. It showed a well-defined, exophytic, lobular lesion with mixed density in proximal jejunum measuring 3 × 4 × 4 cm (Figure 2). The lesion displayed strong arterial phase enhancement, mainly at the periphery, and was supplied by a branch from the superior mesenteric artery and drained by a prominent tributary of the superior mesenteric vein just proximal to the portal venous confluence. Arterial phase enhancement of the draining vein, superior mesenteric (SMV), and portal vein (PV) was indicative of an AVM. The facility for magnetic resonance imaging (MRI) is very limited in our resource-limited setting, and therefore, we did not proceed with an MRI for further delineation of the lesion as the CT images were sufficiently conclusive.

**Figure 3:**
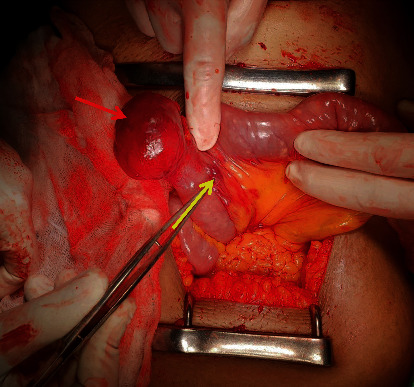
Intraoperative image: red arrow shows the jejunal lesion; yellow arrow shows the main feeding vessel through mesentery.

**Figure 4:**
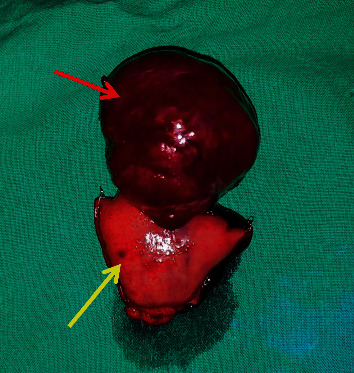
Resected specimen: red arrow shows the jejunal lesion; yellow arrow shows part of the jejunum.

**Figure 5:**
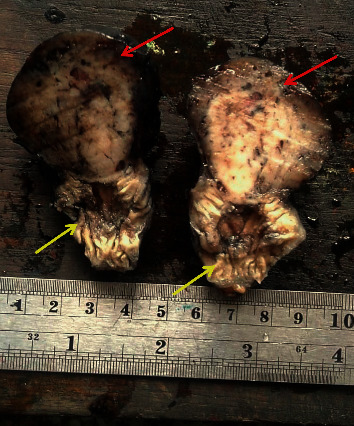
Macroscopic appearance of the tumour cross-section: red arrows show the jejunal lesion arising from the wall; yellow arrows show the jejunal lumen.

Surgery was carried out in the index hospitalization while the patient was stable, in order to avoid a risk of rebleeding. Laparotomy revealed a mass with two prominent feeder vessels at the antemesenteric border of the proximal jejunum, 10 cm distal to the duodenojejunal flexure (Figures 3–5). A segmental jejunal resection including the mass was performed, and continuity was restored with a side to side anastomosis. Histology revealed a gastrointestinal stromal tumour (GIST) composed of short fascicles of spindle cells. There was no histological evidence of an AVM or angiodysplasia. Her postoperative recovery was uneventful. She was referred to the oncologist and received adjuvant oral imatinib therapy.

## 3. Discussion

GISTs occur along the length of the gastrointestinal tract, the stomach, and the small intestine being the commonest locations [7]. Tumour characteristics and behaviour vary between individual neoplasms, and current consensus is based on risk of recurrence and metastasis. Clinical presentation spans from incidental detection to massive gastrointestinal bleeding. The common submucosal location causes overlying mucosal ulceration and bleeding which ranges from occult to life-threatening overt bleeding [8]. The diagnosis relies on contrast CT, endoscopy, and endosonography-guided biopsy [9]. The CT scan finding of a well-defined mass with homogenous contrast enhancement is characteristic of GIST with a sensitivity and specificity of 94.9% and 100%, respectively [10, 11]. However, GISTs mimicking an arteriovenous malformation are uncommon. Imaging alone would not be useful in differentiation, and diagnosing a GIST and magnetic resonance imaging (MRI) scans do not offer any additional benefit [9]. Contrast enhancement of the draining vein of the mass along with the SMV and PV (Figure 2) was suggestive of an AVM. Only 4 such cases have been reported worldwide with similar presentation as summarized in Table 1.

The misinterpretation of AV malformation in our patient was based on the hypervascularity of the tumour with the superior mesenteric vein enhancement in the arterial phase of the CT which was comparable to the contrast enhancement of the superior mesenteric artery and the aorta. Similarly, all reported cases were in the jejunum and CT scans showed contrast enhancement of veins during the arterial phase suggestive of an AVM or angiodysplasia. Three of the reported cases had an associated AVM in histology [3, 4, 6]. Similar to the case reported by Shiozawa et al. [5], there was no histological evidence of an AVM or angiodysplasia in our patient. Vats et al. also reported another patient with a similar presentation found to have a Dieulafoy lesion overlying a gastric GIST without histological features of an AVM or angiodysplasia [12].

Treatment of patients with bleeding GISTs or AVMs depends on the initial presentation and the severity of bleeding. Endoscopic methods are useful in accessible regions, though the rebleeding risk after endoscopic treatment alone is significant, especially in angiodysplastic lesions [13]. In our patient, endoscopic injection of glue allowed stabilisation of the condition before planning surgery similar to angiography and embolization. Surgery was delayed until the CT scan was performed after day 6 of admission as there were logistic issues in performing the CT scan in our resource-limited setting. Differentiation between a vascular malformation and a GIST is useful because segmental resection with clear margins is warranted in all potentially resectable GISTs [14]. However, although important to be differentiated, surgery is also the treatment in the localized AVF involving the bowel.

## 4. Conclusion

We report an unusual presentation of a GIST mimicking an AVM. The possibility of a coexistent or masquerading GIST should be considered when the diagnosis of an AVM is made in patients presenting with gastrointestinal bleeds. In such circumstances, surgical resection and histological confirmation are prudent.

## Figures and Tables

**Table 1:** Summary of previous case reports that described a similar presentation.

	Author	Year of publication	Age (years)	Presentation	Site of GIST	Histology
1.	Tomita et al. [3]	2004	47	Melaena	Jejunum	Submucosal angiodysplasia with spindle cell type GIST
2.	Shim et al. [4]	2008	44	Melaena	Jejunum	Spindle cell type GIST and AVM located in the overlying submucosa and muscularis propria
3.	Shiozawa et al. [5]	2011	62	Intermittent left upper abdominal pain	Jejunum	Spindle cell type GIST without histological features of AVM
4.	Javeed et al. [6]	2015	23	Melaena and anaemia	Jejunum	Spindle cell type GIST and was intermingled with the AVM

## Data Availability

All data generated or analyzed during this study are included in this published article.
